# Genetic characterization of severe fever with thrombocytopenia syndrome virus in ticks from livestock environments in South Korea

**DOI:** 10.3389/fmicb.2026.1936358

**Published:** 2026-09-21

**Authors:** So Youn Youn, Seon Mi Lee, Vinh Hoa Pham, Yun Sang Cho, Mi-Sun Yoo, Dongmi Kwak, Seung-Hun Lee, SungShik Shin, Hyang Sim Lee

**Affiliations:** 1 Bacterial Disease Division, Department of Animal and Plant Health Research, Animal and Plant Quarantine Agency, Gimcheon-si, Gyeongsangbuk-do, Republic of Korea; 2 College of Veterinary Medicine, Kyungpook National University, Daegu, Republic of Korea; 3 College of Veterinary Medicine, Chungbuk National University, Cheongju, Republic of Korea; 4 College of Veterinary Medicine, Chonnam National University, Gwangju, Republic of Korea

**Keywords:** livestock, severe fever with thrombocytopenia syndrome virus, South Korea, surveillance, tick

## Abstract

**Introduction:**

Severe fever with thrombocytopenia syndrome (SFTS), caused by the SFTS virus (SFTSV), is a highly fatal tick-borne zoonosis that poses a substantial public health threat. Although livestock husbandry environments represent important ecological settings for viral maintenance and circulation, molecular surveillance within these settings remains insufficient.

**Methods:**

We conducted an annual systematic entomological and molecular survey from March to October between 2023 and 2025 in South Korea, targeting 204 environments adjacent to cattle, horse, deer, goat, and sheep farms.

**Results:**

Among 101,006 field-collected ticks, *Haemaphysalis longicornis* was the most abundant species (90.62%), followed by *Haemaphysalis flava* (8.20%) and *Ixodes nipponensis* (1.10%). Molecular screening of the SFTSV S-segment revealed 65 positive pools, corresponding to a minimum infection rate (MIR) of 0.06%, with the highest MIR observed in cattle-associated tick populations (0.09%). Phylogenetic analysis demonstrated that all identified strains in this study clustered within the Japanese/Korean clade of genotype B2. Six out of seven contemporary strains shared 100.00% nucleotide identity and were identical to historical domestic human isolates from 2014, demonstrating high longitudinal sequence conservation within the analyzed partial S-segment region. Concurrently, detection of SFTSV strain 24CC401 in *I. nipponensis* larvae provided indirect field-based evidence consistent with potential transovarial persistence. This larval isolate displayed a distinct molecular profile with a 0.65% nucleotide divergence and two nonsynonymous mutations (G129E and V158E).

**Discussion:**

Our findings indicate that specific genotype B2 lineages were detected in livestock-adjacent environments, suggesting a potential ecological association with livestock farming. Although the observed sequence conservation may support continued evaluation of conserved S-segment regions for molecular surveillance, the functional significance of the mutations identified in the larval isolate remains to be determined. Continued molecular surveillance may help characterize SFTSV genetic variation in ticks from livestock-associated environments.

## 1 Introduction

Severe fever with thrombocytopenia syndrome (SFTS) is a burgeoning tick-borne zoonosis caused by the SFTS virus (SFTSV), a member of the *Bandavirus* genus in the Phenuiviridae family. Since its initial detection in South Korea in 2013, SFTS has posed a substantial public health threat due to its high fatality rate and the widespread distribution of its primary vector, *Haemaphysalis longicornis* (Yun et al., 2020). The epidemiological landscape in South Korea is characterized by a disproportionately high case-fatality rate of approximately 18.5%, markedly exceeding those reported in neighboring endemic countries (Korea Disease Control and Prevention Agency (KDCA), 2025). Moreover, the incidence of SFTSV infection increased in 2025, reaching a 5-year peak. This upward trend may be attributable to several factors, including increased outdoor activities and climatic shifts that may enhance the seasonal activity and population density of tick vectors (Yang et al., 2026).

Furthermore, the high fatality rates and evolving clinical manifestations in South Korea are thought to be closely linked to the genetic evolution and diversification of circulating SFTSV strains. The SFTSV genome consists of three negative-strand RNA segments: the large (L), medium (M), and small (S) segments. Genetic reassortments and mutations within these segments—particularly the M segment, which encodes viral glycoproteins—can influence viral entry, pathogenicity, and host immune evasion (Tani et al., 2016; Lee et al., 2023; Wang et al., 2022). Given the high degree of viral mutations and genetic reassortments, continuous genomic monitoring is essential for tracking the evolutionary trajectory of the virus.

Crucially, the risk of SFTSV transmission varies across environments. Unlike general agricultural settings, livestock farming environments provide a unique ecological niche that may facilitate persistent SFTSV transmission. These environments sustain high densities of tick vectors by providing stable blood meals from domestic animals, which may increase the risk of occupational exposure for livestock workers.

Despite the importance of genomic surveillance in these high-risk environments, comprehensive data on the recent genetic characteristics of SFTSV segments circulating within the livestock industry remain limited. To address this knowledge gap, we conducted molecular surveillance of SFTSV in a livestock-associated setting. We genetically characterized partial S-segment sequences originating from ticks collected near animal pens and pastures.

## 2 Materials and methods

### 2.1 Sample collection and species identification

Ticks were collected annually from March to October between 2023 and 2025 across six designated regions of South Korea (the Gangwon, Gyeonggi, Gyeongsang, Chungcheong, Jeolla, and Jeju Provinces), targeting 204 environments associated with cattle, horse, deer, goat, and sheep farms. The collected ticks were examined under a stereomicroscope and identified using morphological criteria for the species and developmental stages, following a taxonomic key (Yamaguti et al., 1971).

### 2.2 Isolation of tick nucleic acids

The ticks were pooled according to species and developmental stage for pathogen detection. The standard pool sizes were 1–50 larvae, 1–10 nymphs, and 1 adult.

The pooled ticks were homogenized with 3 mm stainless steel beads with 500 μL phosphate-buffered saline (Gibco, Waltham, MA, USA) using a Precellys 24 tissue homogenizer (Bertin Instruments, Montigny-le-Bretonux, France). Following homogenization, the tubes were centrifuged at 16,200 × *g* for 1 min, and DNA was extracted using a Maxwell RSC Viral Total Nucleic Acid Purification kit (Promega, Madison, WI, USA) and a Maxwell RSC instrument (Promega) according to the manufacturer's instructions and stored at −70 °C0 °C until further analyses.

### 2.3 SFTSV detection

To detect SFTSV in ticks, we performed nested reverse transcription, polymerase chain reaction (PCR) analysis of the S-segment using a Mastercycler X50 instrument (Eppendorf, Hamburg, Germany; Table 1), as described previously (Yoshikawa et al., 2014). PCR was performed using an AccuPower RocketScript RT-PCR Premix (Bioneer Co., Daejeon, Republic of Korea) for the first reaction and AccuPower ProFi Taq PCR Premix (Bioneer Co.) for the second reaction. PCR was carried out in 20 μL reactions containing 1 μL of 10 nM of each primer, 13 μL of nuclease-free water, and 5 μL of template RNA in a freeze-dried PCR Premix (Bioneer Co.). The amplification conditions for nested PCR were as follows: the first reaction consisted of reverse transcription at 55 °C5 °C for 60 min, followed by initial denaturation at 95 °C5 °C for 5 min, and 30 cycles of 30 s at 95 °C5 °C, 30 s at 52.5 °C5 °C, and 30 s at 72 °C2 °C, with a final extension at 72 °C2 °C for 5 min. The second reaction consisted of initial denaturation at 95 °C5 °C for 5 min, followed by 35 cycles of 95 °C5 °C for 20 s, 55 °C5 °C for 30 s, and 68 °C8 °C for 30 s, with a final extension at 68 °C8 °C for 5 min.

**Table 1:** Primers used for SFTSV detection.

Primers	Sequences (5'–3')	Target gene (bp)	Note	References
NP-2F	CATCATTGTCTTTGCCCTGA	S-segment (461)	RT-PCR	Yoshikawa et al., 2014
NP-2R	AGAAGACAGAGTTCACAGCA			
N2F	AAYAAGATCGTCAAGGCATCA	S-segment (346)	nPCR	
N2R	TAGTCTTGGTGAAGGCATCTT			

### 2.4 Sequencing and phylogenetic analysis

The nested PCR products were analyzed using a microchip electrophoresis system (MCE-202 mULTIna; Shimadzu, Kyoto, Japan). Following electrophoresis, the PCR products were sequenced by Cosmogenetech (Daejeon, Korea). The resulting sequences were compared with previously reported sequences available in the NCBI GenBank database. The SFTSV sequences were aligned using BioEdit version 7.2.5 (Sheng et al., 2025). Subsequently, maximum-likelihood phylogenetic trees were constructed using the Kimura 2-parameter model with 1,000 bootstrap replicates in MEGA6 (Kumar et al., 2016). Pairwise nucleotide identities were calculated using the Create Pairwise Comparison tool with the Percent Identity option in CLC Main Workbench version 20.0.3.

### 2.5 Statistical analysis

Statistical analyses were performed using R. version 4.3.2. Associations between SFTSV detection and tick species, developmental stage, sampling month, and environments associated with farms were evaluated using Fisher's exact test or Chi-square (χ^2^) tests. The 95% confidence intervals (CIs) for MIRs were calculated using the Wilson score method. Statistical significance was set at *p* < 0.05.

## 3 Results

### 3.1 Tick species and prevalence of SFTSV in ticks

To evaluate the distribution and prevalence of potential vectors at the animal–environment interface, we surveyed ticks in 204 areas immediately adjacent to domestic livestock farms, including those rearing cattle, horses, deer, goats, and sheep, using both dragging and flagging methods (Figure 1). We collected 101,006 ticks during the study period. Due to the inherent limitations of morphological diagnostic keys in differentiating immature stages, *Haemaphysalis* spp. larvae were excluded from subsequent species-level classification to ensure taxonomic accuracy. Among the classified specimens that were successfully identified at the species level (*n* = 41,680), *Haemaphysalis longicornis* was the most predominant vector, accounting for 90.62% (37,766/41,680) of the annotated population. *H. longicornis* was followed by *Haemaphysalis flava* at 8.20% (3,417/41,680) and *Ixodes nipponensis* at 1.10% (458/41,680). In contrast, *Amblyomma testudinarium* constituted a minor fraction, representing only 0.09% (39/41,680) of all ticks identified.

**Figure 1:**
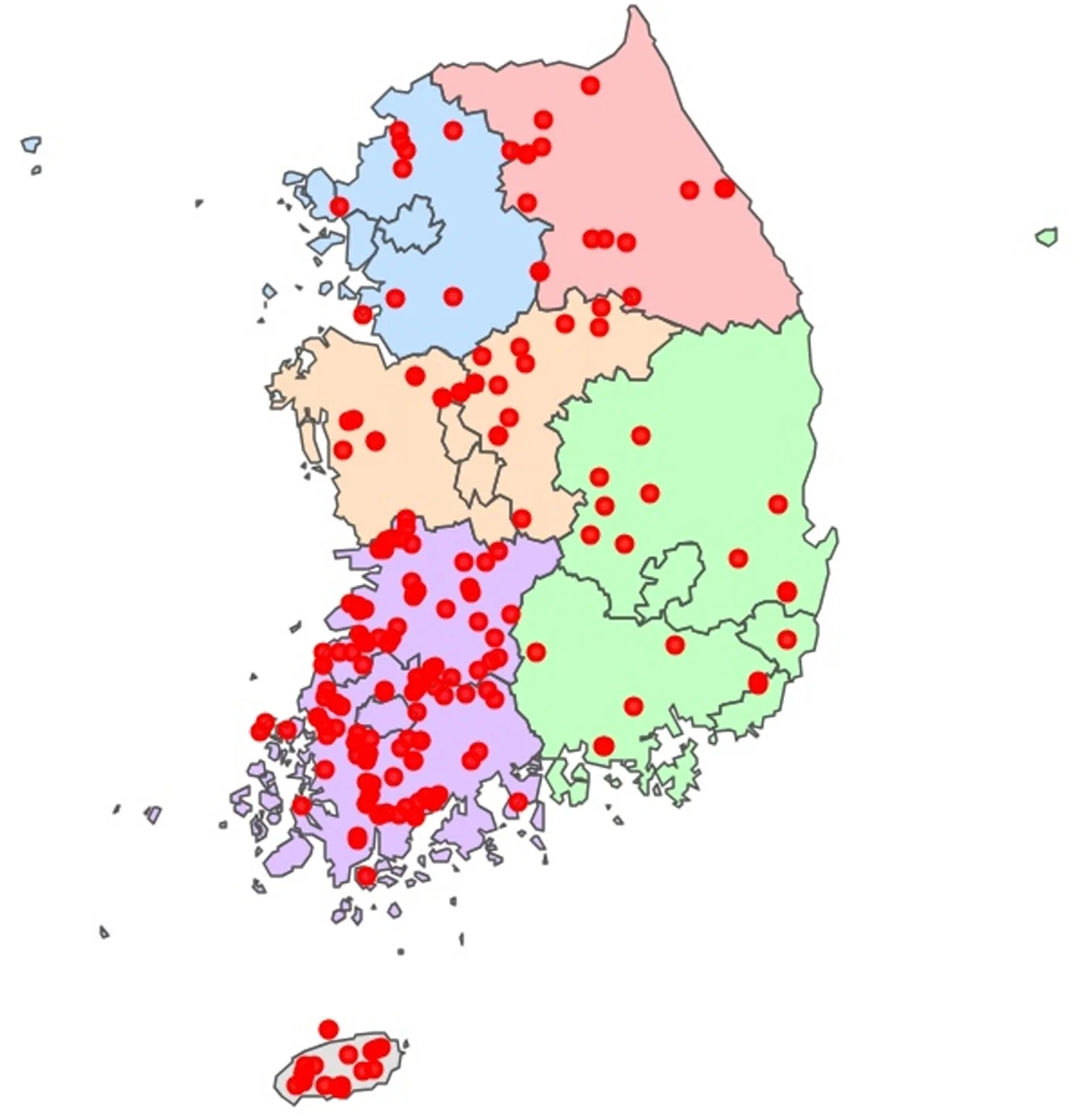
Geographic distribution of the tick-collection sites in South Korea in this study.

### 3.2 Prevalence of SFTSV in ticks across livestock farms

During the three-year surveillance period, 101,006 field-collected ticks were screened for SFTSV, yielding an overall MIR of 0.06% (65 positive pools; Table 2). The MIR exhibited significant variation among tick species (Fisher's exact test, *p* < 0.0001). Among the classified species, *Ixodes nipponensis* displayed the highest MIR (0.22%), followed by *Haemaphysalis longicornis* (0.14%) and *Haemaphysalis flava* (0.12%), whereas no SFTSV was detected in *Amblyomma testudinarium*. Furthermore, MIRs differed significantly across developmental stages (Chi-square test, χ^2^ = 296.38, *df* = 2, *p* < 0.0001), with adult ticks displaying a substantially higher MIR (0.35%) than nymphs (0.12%) and larvae (0.02%) (Table 2).

**Table 2:** Prevalence of SFTSV in ticks collected in South Korea.

Tick species	Number of tested ticks (Adult/nymph/larva)	Number of positive pools^*^ (MIR%^#^)				95% Cl^**^
		Adult	Nymph	Larvae	Total	
*H*. *longicornis*	37,766 (2,490/35,276/0)	10 (0.40)	42 (0.12)	–	52 (0.14)	0.10%−0.18%
*H*. *flava*	3,417 (576/2,841/0)	2 (0.35)	2 (0.07)	–	4 (0.12)	0.04%−0.30%
*I*. *nipponensis*	458 (340/93/25)	–	–	1 (4.00)	1 (0.22)	0.04%−1.23%
*A*. *testudinarium*	39 (2/34/3)	–	–	0	–	0.00%−9.03%
*Haemaphysalis* spp.	59,326 (0/0/59,326)	–	–	8 (0.01)	8 (0.01)	0.01%−0.03%
Total	101,006 (3,408/38,244/59,354)	12 (0.35)	44 (0.12)	9 (0.02)	65 (0.06)	0.05%−0.08%

^*^Collected ticks were pooled, each pool consisted of 1 adult ticks, 1–10 nymphs, or 1–50 larvae, and were used for the detection of SFTSV.

^#^ MIR%: minimum infection rate, (number of positive pools/total number of tested ticks) × 100, ^**^Statistically significant differences in infection rates were evaluated across tick species using Fisher's exact test (*p* < 0.0001) and across developmental states using the Chi-square test (χ^2^ = 296.38, df = 2, *p* < 0.0001). 95% confidence intervals (CIs) were calculated using the Wilson score method.

Seasonal-dynamics analysis revealed that SFTSV infection rate, as quantified by the MIR, exhibited significant monthly variation (Chi-square test, χ^2^ = 82.26, *df* = 7, *p* < 0.0001). Although the number of positive pools displayed a prominent bimodal distribution pattern, the MIR peaked sharply during early spring, reaching its maximum in March at 0.31% (95% CI: 0.17%−0.56%), followed by April (0.14%, 95% CI: 0.09%−0.22%) and May (0.13%, 95% CI: 0.08%−0.21%), before exhibiting a progressive decline throughout the summer. This downward trajectory led to a low MIR in July (0.05%, 95% CI: 0.02%−0.13%). Although the number of positive pools increased slightly in August (*n* = 6) and September (*n* = 9), the MIR remained consistently low in August (0.04%, 95% CI: 0.02%−0.09%) and September (0.04%, 95% CI: 0.02%−0.08%) during this early autumn period, before decreasing to a near-undetectable level in October (0.004%, 95% CI: 0.00%−0.02%) as tick activity substantially declined (Figure 2).

**Figure 2:**
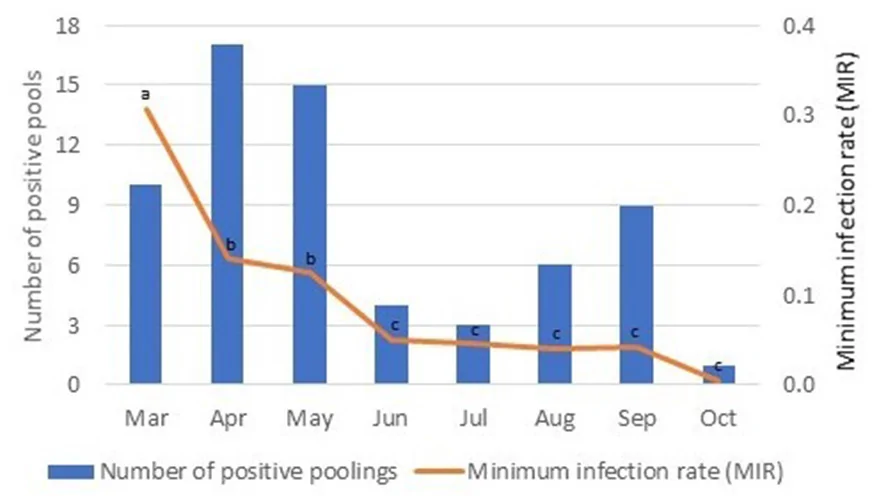
Monthly distribution of SFTSV-positive pools and MIRs during tick surveillance from March to October. Different lowercase letters (a–c) above the MIR line indicate statistically significant monthly differences based on *post-hoc* pairwise comparisons (*p* < 0.05, chi-square test).

Host-specific analysis revealed that the SFTSV infection pressure (MIR) exhibited significant variation across different livestock farm environments (Chi-square test, χ^2^ = 23.86, df = 4, *p* < 0.0001). The MIR was highest in tick populations sampled in the vicinity of cattle farms at 0.09% (95% CI: 0.07%−0.12%), followed by deer farms (0.05%, 95% CI: 0.02%−0.11%), horse farms (0.03%, 95% CI: 0.02%−0.06%), and goat farms (0.03%, 95% CI: 0.01%−0.08%). In contrast, no SFTSV was detected in tick populations collected from the vicinity of sheep farms (95% CI: 0.00%−0.16%); (Figure 3).

**Figure 3:**
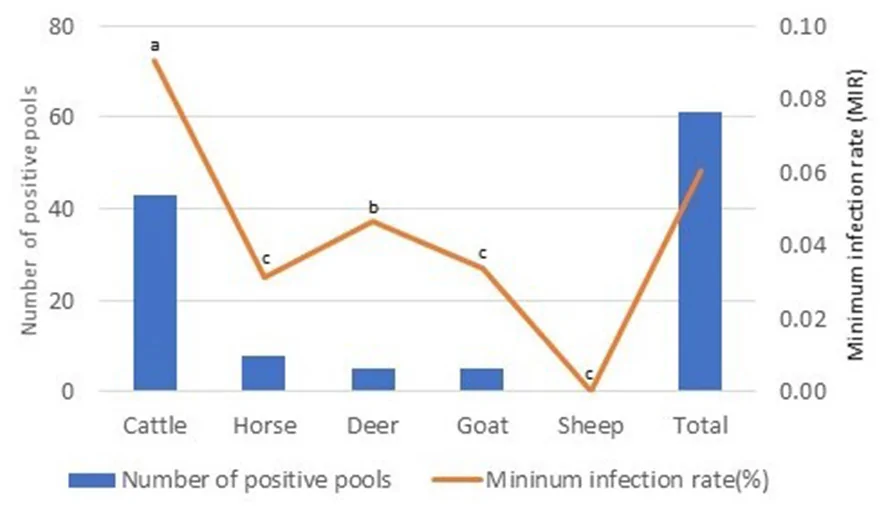
Comparison of SFTSV-positive pools and MIRs in tick populations across different livestock farm environments. Different lowercase letters (a–c) above the MIR line indicate statistically significant differences in SFTSV infection rates among livestock farm environments based on *post-hoc* pairwise comparisons (*p* < 0.05, chi-square test).

### 3.3 Genotypes of SFTSV from ticks circulating from 2023 to 2025

Phylogenetic analysis based on the partial S-segment revealed that all sequenced SFTSV strains from this study clustered exclusively within genotype B2, alongside previously reported reference strains (Figure 4). Within the identified genotype B2 cluster, further sequence analysis revealed that the nucleotide sequences of six of the seven analyzed strains were completely identical, with the sole exception of strain 24CC401 (GenBank accession number: PZ673912). A highly consistent pattern was observed in the deduced amino acid sequences. The six identical strains exhibited completely conserved amino acid sequences, whereas strain 24CC401 displayed two non-synonymous mutations, specifically G129E and V158E (Figure 5). The partial nucleotide sequences generated in this study were deposited in the GenBank database under accession numbers (PZ673908–PZ673914).

**Figure 4:**
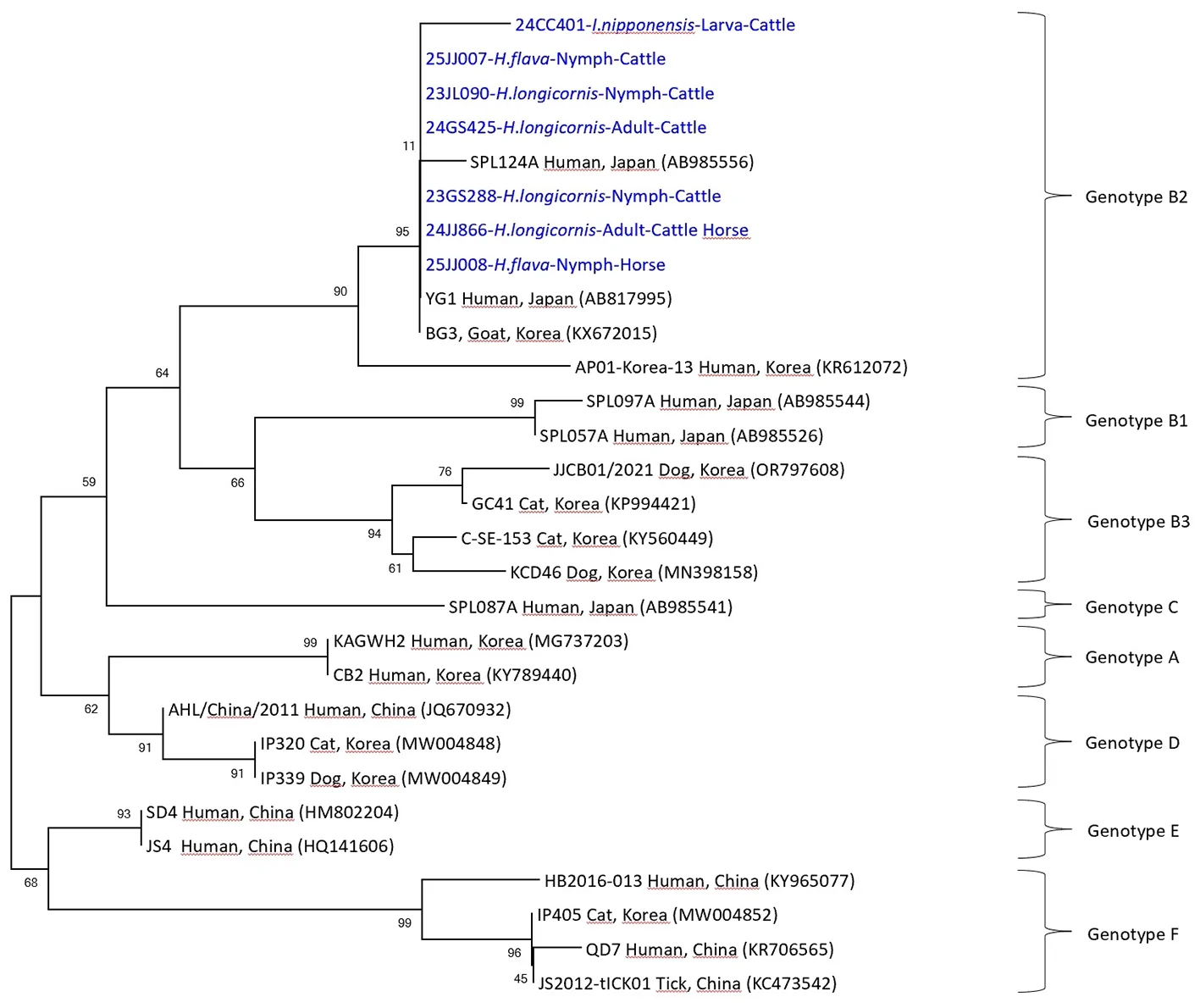
Phylogenetic tree of SFTSV S-segment sequences obtained in this study (indicated using blue letters) and reference sequences retrieved from GenBank (labeled with their accession numbers). Genotypes (A–F) and subclades within genotype B (B1–B3) are shown on the right. Numbers at the nodes indicate bootstrap support values based on 1,000 replicates.

**Figure 5:**
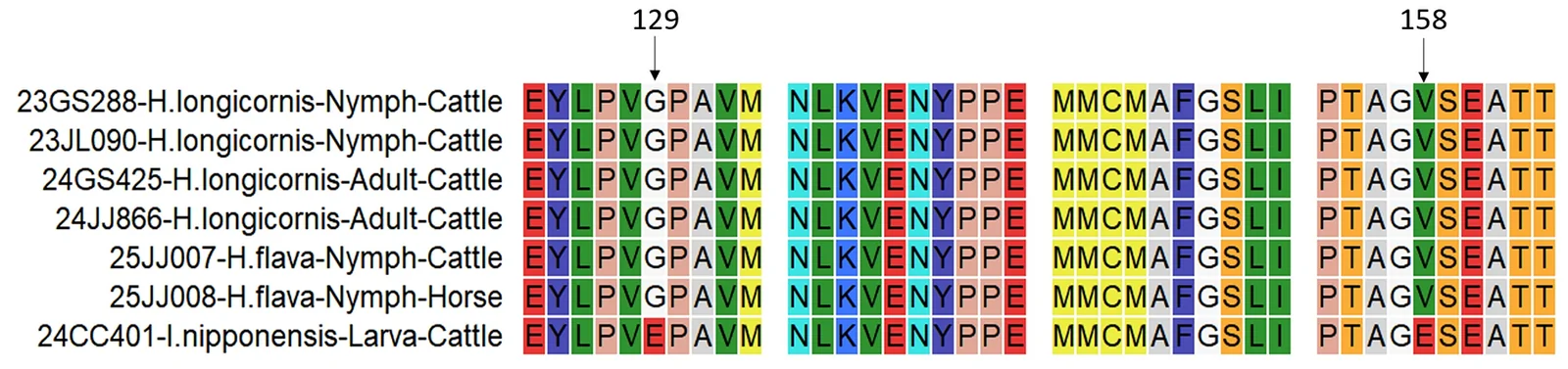
Multiple sequence alignment of amino acid sequences from SFTSV strains obtained in this study.

### 3.4 Geographical divergence within SFTSV genotype B

To elucidate the phylogeographic relationships of the newly identified SFTSV strains, a comparative phylogenetic analysis was performed alongside nine representative GenBank reference sequences of genotype B, including five strains from China and three from Japan. The resulting phylogenetic topology demonstrated a clear geographical bifurcation within genotype B, with all strains identified in this study clustering exclusively within the Japanese/South Korean clade rather than the Chinese lineages (Figure 6). This clustering pattern is consistent with the reported geographical distribution of SFTSV genotypes across East Asia, where genotype B2 predominates in Japan and South Korea, whereas China is characterized by genotypes B1(KR017822) and B3 (JX462445, KF374683, KR017826, PQ469750), with genotype B2 appearing to be comparatively uncommon (Yun et al., 2020). The close phylogenetic affinity between our domestic livestock-associated isolates and strains circulating in Japan indicates high degree of nucleotide similarity and supports regional circulation of this lineage across the Korean Peninsula and the Japanese archipelago.

**Figure 6:**
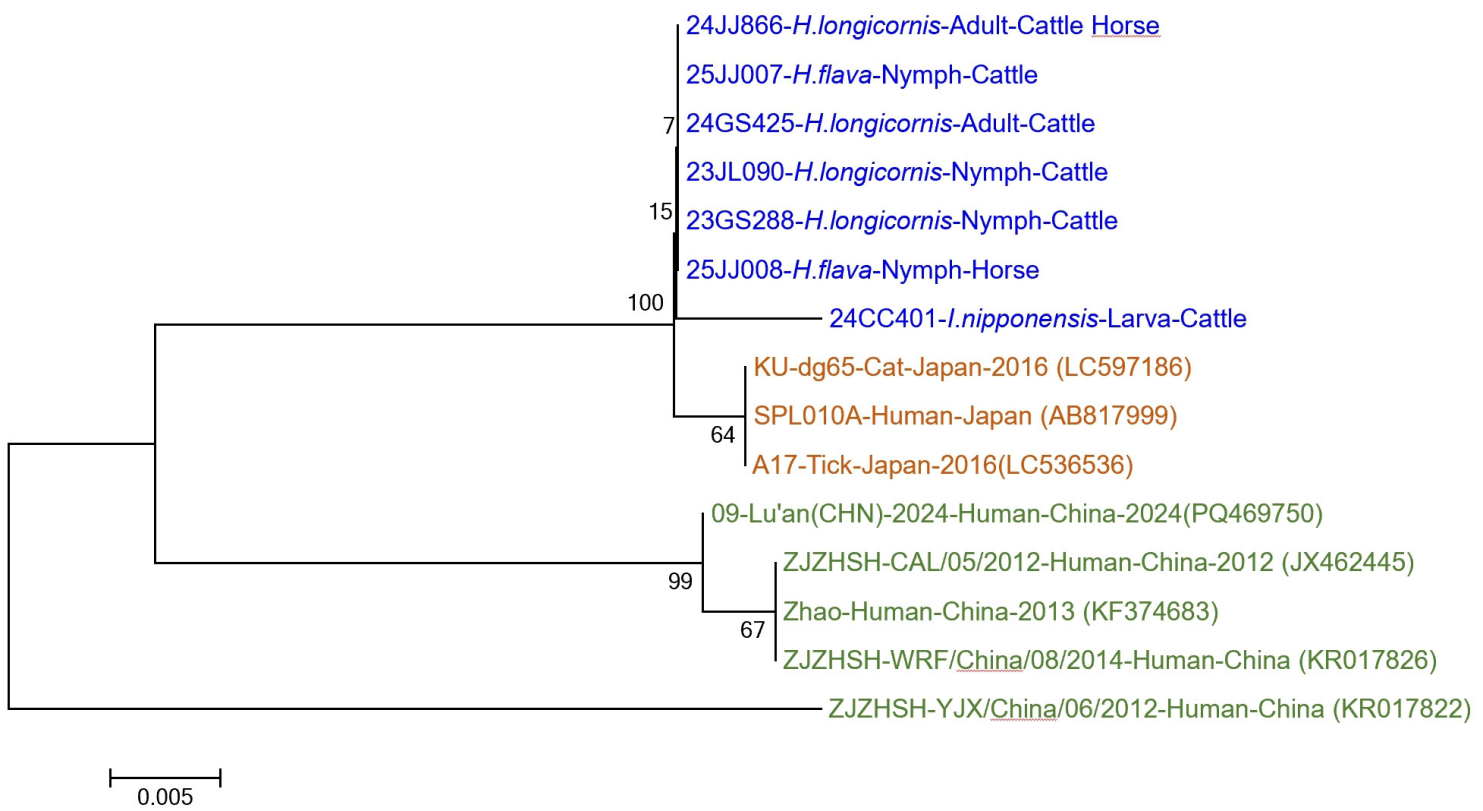
Phylogenetic topology and genotype B2 phylogeographic dynamics of SFTSV strains detected in this study. The strains detected in this study are marked in blue, the Chinese strain is marked in green, and the Japanese strain is marked in orange, with their respective host origins and GenBank accession numbers indicated. Bootstrap support values from 1,000 replicates are presented at the tree nodes.

### 3.5 Phylogenetic and host-specific analysis of genotype B2 isolates

We conducted comparative molecular analysis within genotype B2 for the seven domestic SFTSV strains identified between 2023 and 2025 in this study (23GS288, 23JL090, 24GS425, 24JJ866, 24CC401, 25JJ007, and 25JJ008); (Figure 7). The phylogenetic topology revealed that these isolates shared an extremely close genetic lineage with established pathogens currently circulating within domestic livestock. When categorized by host species, our strains exhibited tight evolutionary clustering with strains derived from livestock, specifically cattle (OR989754) and goats (KX672015). These tick-derived isolates showed closer phylogenetic relatedness to livestock-associated strains than to strains derived from companion animals, such as dogs (MW004851 and MW004854) or cats (LC597186). In contrast, the domestic isolate detected in 2020 (OR987951 and OR987952) clustered within a distinct genotype B2 sublineage together with companion animal-derived strains, indicating that genotype B2 comprises multiple phylogenetically distinct sublineages circulating in South Korea. These findings suggest that the recent livestock-associated genotype B2 lineage may represent a distinct circulating sublineage, whereas the earlier lineage may represent a separate phylogenetic lineage, highlighting temporal differences in the distribution of genotype B2 sublineages rather than strict host-specific clustering.

**Figure 7:**
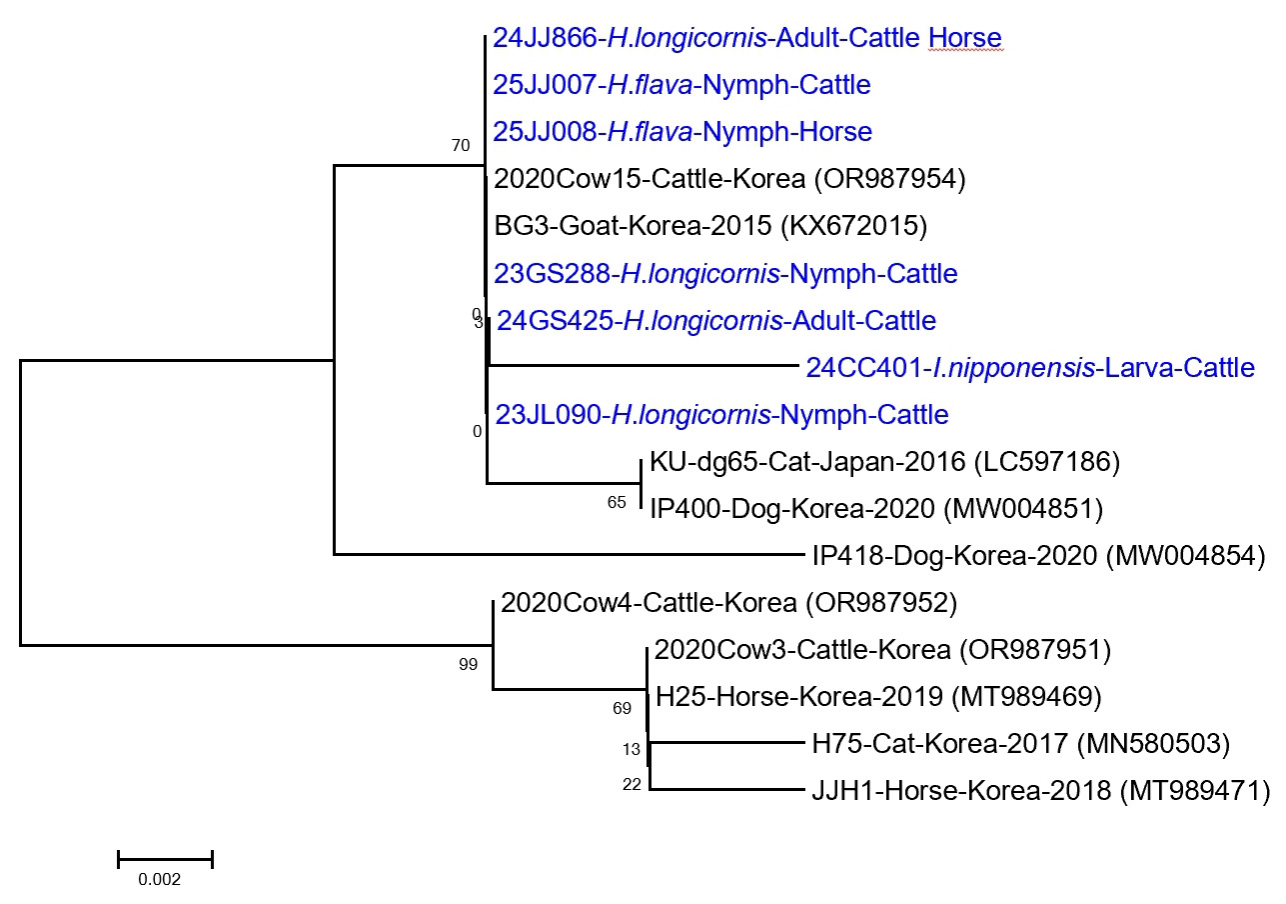
Host-associated phylogenetic analysis of SFTSV S-segment sequences from various animal species. Sequences obtained in this study (blue) are compared with GenBank reference strains labeled with their host species, country, and accession numbers. Bootstrap support values based on 1,000 replicates are indicated at the nodes.

### 3.6 Longitudinal genetic stability of SFTSV S-segment

Between 2023 and 2025, SFTSV strains were detected in multiple tick species (*H. longicornis, H*. *flava*, and *I*. *nipponensis*) within domestic livestock farm environments in South Korea. These strains exhibited high genetic homogeneity and remarkable longitudinal stability (Figure 8). Nucleotide sequence alignment of the S-segment revealed that six strains, spanning 2023 (23GS288, 23JL090), 2024 (24GS425, 24JJ866), and 2025 (25JJ007, 25JJ008), shared 100.00% nucleotide identity, despite variations in the sampling year, geographical location, and associated livestock hosts. Comparative analysis with historical domestic human reference strains isolated from 2013 to 2020 showed that these six contemporary tick-derived strains were completely identical (100.00% homology) to the 2014 human isolates KAGWH3 (KP663736) and KAGBH5 (KP663739). This exceptionally high conservation indicates a high degree of sequence conservation within the analyzed partial N gene locus.

**Figure 8:**
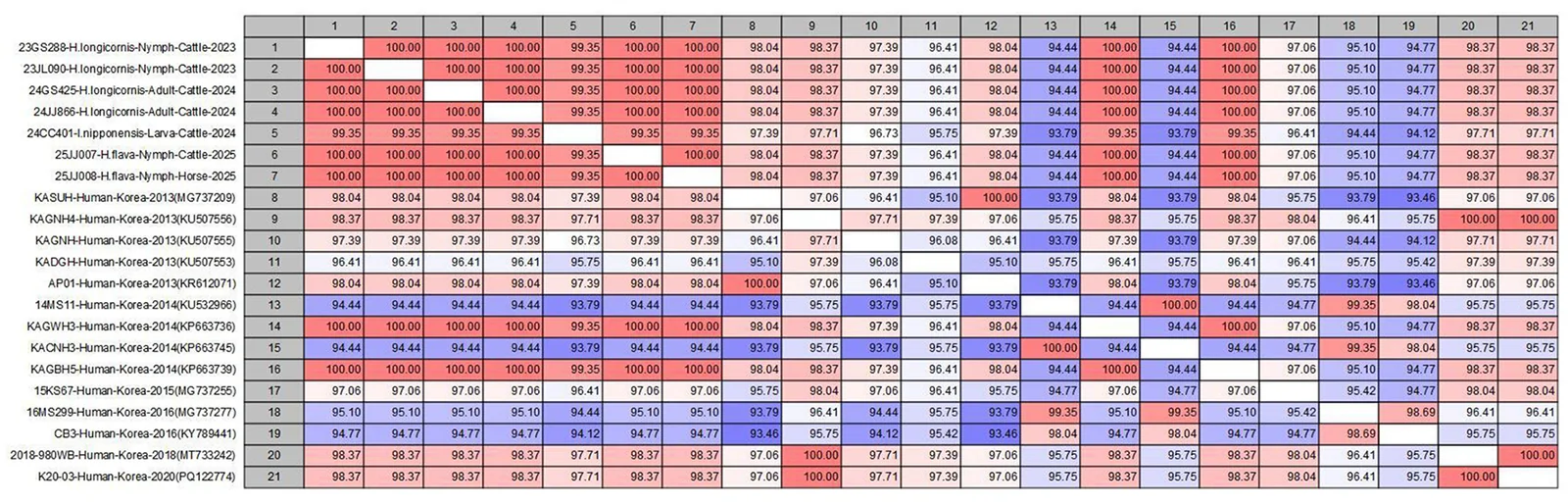
Pairwise genetic distances (substitutions per site) among SFTSV S-segment sequences, calculated using the Kimura 2-parameter model.

Conversely, strain 24CC401, retrieved from an *I*. *nipponensis* larva in 2024, displayed a distinct molecular signature, exhibiting 99.35% identity to the other six contemporary isolates and the 2014 reference strains. The detection of SFTSV in a larval-stage tick provides indirect field-based evidence consistent with potential transovarial transmission. Furthermore, the observed nucleotide divergence (0.65%) in this larval isolate indicates limited sequence variation within the analyzed region.

## 4 Discussion

The systematic tick-borne and molecular surveillance conducted in this study underscores the role of livestock-associated environments as key ecological interfaces for SFTSV detection. Screening field-collected ticks from areas adjacent to domestic livestock farms revealed a higher MIR in tick populations near cattle farms. This high pathogen load at the animal–environment interface aligns with the One Health framework and identifies pastoral ecosystems as high-risk zones for vector-borne zoonoses (WHO et al., 2019; Chaorattanakawee et al., 2022).

The elevated MIR observed during March (0.31%) highlights the substantial public health risk posed by overwintered nymphs and adults. Although these stages are less abundant in the field than are summer larvae, their higher MIRs underscore an enhanced viral reservoir capacity, likely driven by successful transstadial maintenance or prior-year amplification. Conversely, the sustained low-baseline MIR observed in August and September offers important empirical evidence regarding vertical transmission. Despite the substantial seasonal emergence of newly hatched larvae lowering the relative pool: sample ratio, the persistence of a stable MIR during this window demonstrates that transovarial transmission is sufficiently robust to maintain a steady baseline of infection within the nascent vector cohort, ensuring continued circulation of B2 genotype sublineages within livestock environments before winter diapause.

Our findings also demonstrate high sequence conservation among SFTSV strains detected in ticks from domestic livestock farm environments. Our partial S-segment analysis revealed that six out of seven strains from 2023 to 2025 were identical at the nucleotide level and exhibited 100.00% nucleotide identity with historical domestic human isolates (KAGWH3 and KAGBH5) from 2014. This longitudinal S-segment sequence conservation indicates a high degree of sequence similarity among the analyzed SFTSV genotype B2 strains. The observed sequence conservation across strains sampled between 2023 and 2025 and the 2014 human reference strains demonstrates conservation within the analyzed partial S-segment region.

In our phylogeographic analysis, the isolates demonstrated clear geographical bifurcation within genotype B, clustering exclusively within the Japanese/Korean clade rather than within the Chinese clade (Yoshikawa et al., 2015). This topological affinity is consistent with the reported geographic distribution of genotype B in East Asia (Yun et al., 2020). Furthermore, the close genetic contiguity observed between our tick-derived strains and established reference strains from domestic cattle, goats, and horses—coupled with a substantially lower affinity toward domesticated companion animal strains—is consistent with a specialized sylvatic and domestic transmission cycle (Niu et al., 2013; Chae et al., 2024). These results highlight the phylogenetic diversity of genotype B2 lineages associated with livestock farms, potentially reflecting transmission patterns distinct from those involving companion animals.

The 24CC401 strain from an *I. nipponensis* larva collected in 2024 provides key insights into the localized evolutionary dynamics of SFTSV. Although the core genome of the dominant B2 lineage remained highly conserved, this larval isolate displayed a subtle yet distinct molecular signature, characterized by two nonsynonymous mutations (G129E and V158E). The detection of SFTSV in a larval tick provides indirect field-based evidence consistent with potential transovarial (vertical) persistence within the South Korean vector population (Park et al., 2016). The observed nucleotide divergence in the 24CC401 strain suggests that, although vertical transmission ensures the baseline persistence of the virus in the absence of amplifying hosts, transovarial passage or host-specific adaptation might introduce specific selection pressures, inducing subtle genetic drift (Zhuang et al., 2018).

We acknowledge the inherent limitations of utilizing partial S-segment nucleotide sequences rather than whole-genome sequencing. Given the segmented nature of the SFTSV genome, whole-genome sequencing or sequencing of the M segment would be valuable in future studies to evaluate potential genetic reassortment and glycoprotein variation across the L, M, and S segments, which frequently drive the emergence of novel phenotypic variants (Lee et al., 2023). Nevertheless, the partial S-segment target employed herein offers substantial diagnostic and molecular value; it encapsulates highly informative antigenic determinants and represents one of the most reliable loci for genotype classification and phylogeographic benchmarking (Wang et al., 2023; Sang et al., 2024). Thus, our findings provide a baseline for assessing longitudinal S-segment sequence conservation, with field observations consistent with potential transovarial transmission ecological.

From a public health and veterinary perspective, this molecular analysis has critical implications for developing medical countermeasures. Although no globally approved commercial SFTSV vaccines are available for humans or livestock, several vaccine candidates, including mRNA- and DNA-based platforms, have shown promise in preclinical studies (Kim et al., 2024). The sequence conservation observed among the dominant domestic B2 strains over the past decade supports the continued evaluation of conserved S-segment regions as potential targets for molecular surveillance. However, the implications of this sequence conservation for viral phenotype or vaccine performance remain to be determined through functional studies. Conversely, the discovery of mutations (G129E and V158E) in the larval strain 24CC401 highlights the value of continued molecular surveillance. If these amino acid substitutions occur within functionally or antigenically relevant regions, their potential effects on antigenicity, diagnostic performance, or vaccine-mediated neutralization warrant further investigation (Li et al., 2012). To further characterize SFTSV genetic variation, high-throughput molecular genetic monitoring may be useful for surveillance of ticks in livestock farm vicinities and clinically symptomatic livestock hosts.

## Data Availability

The datasets presented in this study can be found in online repositories. The names of the repository/repositories and accession number(s) can be found in the article.
